# Joseph K. visits the sick house: how the medical humanities require the medical posthumanities

**DOI:** 10.1007/s40592-025-00242-5

**Published:** 2025-04-25

**Authors:** Martin J. Fitzgerald, Peter J. Katz

**Affiliations:** 1 Department of Biomedical Education and Anatomy, Center for Bioethics and Medical Humanities, College of Medicine, The Ohio State University Wexner Medical Center, Columbus, USA; 2 University Hospitals Cleveland Medical Center, Cleveland, USA

**Keywords:** Medical humanities, Narrative ethics, Posthumanism, Disability

## Abstract

This paper challenges the typical function of narrative in the medical humanities to advocate for a medical *post*humanities: an approach that destabilizes the centrality of "the human" and instead embraces patient narratives that are embodied, fragmented, and provisional. To make this claim, we first challenge the stability of the “humanity” described in the “medical humanities” and reiterated in the genre that we call “the medical romance.” In this genre, illness and suffering destabilize a sense of identity and coherence, which is then restored through introspection and interpretation of the patient narrative. To challenge this genre, we turn to *surface reading*, a literary studies technique that sees traditional interpretation as too hurriedly foreclosing on meaning. Through a close reading of Franz Kafka’s *The Trial *and Henrik Ibsen’s *Hedda Gabler*, we demonstrate both what surface reading looks like and also how it embraces generic and interpretive instability. Finally, we focus this approach to narrative on physician-assisted suicide (PAS), particularly attending to PAS and disability, to argue that both medical romance and its entailed traditional narrative interpretation overvalue “the human” as an agential individual seeking a “good death.” This at once affirms the tendency to encourage the allegedly meaningful death of disabled people by PAS, and also excludes from narrative focus the structural and environmental sources of suffering. The medical posthumanities, in its attention to embodiment, networks, environment, and the decentralizing of individual agents, would better make room for patient narratives that value the messiness and interconnectedness of lived experience.

## Introduction

When we look at the medical humanities, what do we see? The field is certainly disparate in orientation and composition. At the very least, it is clear that there are multiple different strands of inquiry and practice that happen to march together under the shared banner of the medical humanities. However, it is yet unclear the extent to which these strands come together to form a true coalition, or whether they merely ensnare one another. The line between an “anarchic” (Kidd and Connor 2008) and a cross-/inter-/multi-/disciplinary approach may not be a particularly luminous one. Institutionally, the medical humanities are nomadic; the field can be found in a variety of different disciplinary homes. For instance, at The Ohio State University (the institution of one of this paper’s authors), there is both the Center for Bioethics and Medical Humanities housed within the Department of Biomedical Education and Anatomy within the College of Medicine, as well as an MA in the Medical Humanities housed within Department of English within the College of Arts and Sciences. It is sensible, then, that this journal is now asking where the medical humanities currently stand and where they ought to go.

Much as modern bioethics has hung its hat on the concept of autonomy, so have the modern medical humanities rallied around the importance of narrative. In this paper, we will use narrative as a means to demonstrate how, to fulfil its task, the medical humanities will ultimately require the medical *posthumanities*, by which we mean a vision of the humanities which overcomes arbitrary (which is not to say *all*) centering of the human in meaning-making (Welch 2024). Narrative medicine, as it is currently practiced, often takes the generic conventions of medical *romance*, whereby an ailing patient is expected to stitch together their experiences so as to reassert their own personal identity over and against their sickness. Although there is certainly something interesting about medical romance as a genre, it is also an interpretive frame which forecloses on alternative modes of meaning-making. To demonstrate this, we will begin with an overview of the current state of the medical humanities. From there, we will detail what we mean by the term “medical romance.” We will use Kafka’s *The Trial* to reveal deeper aspects of narrative, such as the need for so-called “zones of indifference” and “zones of interest.” Finally, we will use these conceptual tools to analyze how unquestioned deployment of medical romance constrains analysis of physician-assisted suicide in disabled patients. We take this as evidence of the importance of a medical *post*humanities which operates in light of the undecidability and interminability (though not the *relativism*) of interpretation by decentering the human from its default position as the measure of all things.

## Where bioethics and the medical humanities currently lie

The medical humanities, as they currently stand, are several. As Bleakley summarizes:

“The descriptor ‘medical humanities’ has been applied to the following five fields of activity:

- The humanities studying medicine (such as history of medicine or the critical evaluation of medicine in literature).
- Arts and humanities intersecting with medicine in medical education – often called ‘medicine as art’.
- The arts engaging with medical themes in public engagement.
- Arts for health (for example, art in hospitals and arts activities with patients – often called ‘arts as medicine’).
- Arts therapies (sometimes linked with arts for health, but usually associated with mental health interventions using arts media within a psychotherapeutic framework)” (Bleakley 2015, p. 45).

Although these five fields may overlap in places, clearly these are not straightforwardly the same thing. If this is the case (and it surely seems to be), then one must tread carefully before delimiting the scope of the field. After all, to say that the goal of the medical humanities is to aid in the realization of desirable medical outcomes via the use of the arts,^1^ would apply quite well to art therapy but would apply only very obliquely indeed to artists who take healthcare as major inspiration for their work.

Thus, to begin, we need to get clear on our portrait of the medical humanities. Our approach sees the genesis of the field precisely in the meeting of the two concepts which compose it: medicine and the humanities. As to the humanities aspect,

The concept of ‘the humanities’ focuses on studying human experience: what can happen to people and what people can do; possible ways of thinking, ways of feeling, and ways of speaking; possible motives and possible values. The words *can* and *possible* highlight the imaginative character of the research in ‘the humanities.’ They also highlight the double focus of ‘the humanities’: on ‘humanity’ as a whole and on individual (though culturally embedded) human beings in all their immense diversity. (Wierzbicka 2011, p. 36. Emphasis in the original).

The humanities study human experience, whether that be at the level of an individual human or at the level of humanity taken as a whole. Many authors (including Wierzbicka herself) take this to be a task that has an inherently normative edge.

What, then, makes the humanities into the medical humanities? On Evans’ telling

the medical humanities are just those same studies concerned with the specific experiences of health, disease, illness, medicine and health care, with the practitioner–patient relationship and, above all, with the clinical consultation as a focal arena for such experiences” (Evans 2002, p. 510).

They are indeed what the name promises: the humanities as they enter into the realm of medicine. The various disciplines that constitute the field such as literary studies, history, philosophy, religious studies, and so forth, can be brought to bear on the task of studying human experience within this context. Within the medical humanities, there is a particular value in then turning that study back towards deployments within medicine itself, such as in the case of using the medical humanities in medical education (Smydra et al. 2022).

However, importantly, one must note that the humanities rely on the idea of the human. As Foucault aptly notes, the human is neither an uncontested nor a self-evident concept (Foucault 2005). Rather, its meaning must itself be born out in investigation. This is, though, not a straight-forward suggestion. If we are humans asking questions about ourselves and our experiences, and what we decide characterizes our experiences ends up altering how we view ourselves as humans, then the very questions we ask are implicated (and possibly dramatically altered) in the answers we are giving. The dilemma is a case of Gabriel Marcel’s notion of mystery, whereby:

A problem is something which I meet, which I find completely before me, but which I can therefore lay siege to and reduce. But a mystery is something in which I am myself involved, and it can therefore only be thought of as a sphere where the distinction between what is in me and what is before me loses its meaning and initial validity (Marcel 1949, p. 117).

Said another way, a mystery is a “problem that encroaches on its own data” (Marcel 1995, p. 19). So, what, then, can we say about the human?

We can begin with an eye towards the human as related to narrative; one of the medical humanities’ perennial bailiwicks is patient/provider narratives. Indeed, one of the core vehicles for medical humanities is case study analysis, where case studies themselves are narrative presentation. To modify Arthur Frank’s notion of the “wounded storyteller,” (Frank 2013) we can then begin with the human as a *woundable* storyteller. In other words, as a reprise of Evans, “the point of view of frail, flesh-bound, human experience” (Evans 2002, p. 501) is the point of view of a woundable storyteller. It is the point of view of a being that can make narratives about itself and does so as embodied and radically open to the world.

## Narrating the medical romance: narrative, interpretation, and genre

Narrative sits at the center of much medical humanities work. Hunter (1991) described physicians’ diagnostic process as a kind of detective work on patient’s stories: the interpretation of patient narratives through the lens of their own medical knowledge. Rita Charon’s “Narrative Medicine” (2001a) and “The Patient–Physician Relationship” (2001b), and the subsequent work she and her colleagues at Columbia developed, shaped the conversation about narrative in medical humanities at the turn of the century as she advocated for “narrative competence,” or “the ability to acknowledge, absorb, interpret, and act on the stories and plights of others” (2001b, p. 1897). In this section, we will argue that while this approach certainly has valuable effects, the understanding of narrative in the medical humanities has been largely limited to narratives about the preservation or reformation of personal identity, or what we will call the *medical romance*. These narratives foreclose on the affective and narrative possibilities for stories of illness, of being a patient, of being a healthcare worker. But this is an issue of theory as much as it is genre.

Over the past few decades, the vast majority of narrative medicine has focused on a narrative structure that is fundamentally *romantic*, generically: illness disturbs the equilibrium of a patient or physician’s identity; they tell their story as a way to grapple with those changes and shifts; the story concludes with a return to self, perhaps changed, but ultimately improved. Even if the patient succumbs to their illness, in terms of a physician narrative, the tragedy improves the physician (and those around) the patient *as humans*. Martignoni et al (2012) call on the medical humanities as a way to “address the vulnerability of man [sic]” (p. S2). They write that, “[a]t the center of the Medical Humanities, there is self-narration, telling one’s story, about one’s suffering, the anguish and restlessness,” and that telling these stories can help repair the “existential damage” that illness inflicts (p. S2). Chiavaroli (2017) celebrates how “specific activities involved in the study of literature, such as close reading, critical reflection and ‘countercultural perspectives,’ have recently been linked to the process of professional identity formation” (p. 19). The work of narrative, according to these approaches, is instrumental (Bleakley and Nielsen 2024, pp. 5, 8) in the vein of Montgomery Hunter’s detective-doctors: narrative as a vehicle to make order out of chaos, to derive conclusions from complexity. It will make good healthcare practitioners and good patients by pinning down a meaningful sequence of events that culminates in the restoration of the human.

While romantic “restitution narratives” are lovely, as Shapiro et al (2009) observe, their centrality in the medical humanities renders “all other narratives … synonymous with failure” (2009, p. 196). Intrinsically, the restitution narrative aims to structure uncertainty, to return identity and society back to normal (Thacker et al. 2022, p. 327). Even if the disease remains untreated or uncured, the physician is meant to reiterate that humanity perseveres and is restored. Narrative medicine in this mode “exaggerate[s] the coherence and elaborateness of everyday illness narratives” (Kirmayer 2024, p. 23; see also Kirmayer 2000 and Woods 2011). The medical romance compels generic structure and coherence, culminating in a conclusion that produces what Fitzgerald and Collard (2016) refer to as “those wearyingly familiar encomia – an ‘ethical life’ and a ‘good death’ ” (p. 44). Conclusions, especially when shaped by generic conventions, will reinforce typical restorations of the status quo, and rein in deviations from the genre—and the cultures that reinforce that genre (Katz 2022, p. 161).

And in the case of the medical humanities, “the human” is that thing which is conserved. A medical posthumanities would “actively reflec[t] upon and interrogat[e] the normative and individualist restrictions” entailed in the narrative traditions so central to the medical humanities (Viney et al. 2015, p. 2). As Bishop (2008) rightly observes, Charon’s work depends implicitly on:

A sense that the being of humans is the production of language, and in language what is produced is meaning. The act of writing itself, the narrative act, is a production that invests the biological—that is to say, the really real part of a human being—with meaning (p. 20).

If humans are “the storytelling animal” (MacIntyre 2007, p. 201), narrative and language would seem to separate out the humanness from the merely biological, the merely animal (Bishop 2008, p. 16). Fundamentally, as Bishop observes, in the humanist narrative *meaning* distinguishes mere animality from the complexity of humanity.^2^ And in the medical humanities and much of literary studies, *meaning* emerges from *interpretation*—from seeing what is behind the surface. In these contexts, interpretation has become conflated with or supersedes the other elements of narrative; as Charon writes, narrative is meant to hone “the ability to acknowledge, absorb, interpret, and act on the stories and plights of others” (2001b, p. 1897). That is, to put it uncritically, a whole lot for narrative to carry.

But this is an anemic—if dominant—understanding of narrative. In the arc of his work, Alan Bleakley has made a case against the hegemony of narrative and how it “overla[ys]” patients’ stories with plot “to make sense of the patients account” at the cost of distorting patients’ lived experience (Bleakley and Neilson 2022, p. 12). The patient–doctor relationship, when forced through the strainer of narrative, becomes an “agreed story” (Warmington 2020)—one permeated and desiccated by “tired, even dead, metaphors” (Bleakley and Neilson 2022, p. 15; Bleakley 2017, p. 28). These metaphors are too easily reduced to temporalized steps from chaos to ordered meaning-making: [cancer = war] allows the oncologist a ready (if brittle) narrative framework to cast the battle their patient faces (Sontag 1978). Narrative as a temporally charted series of dead metaphors are part of the “habitual, de-sensitizing pedagogical process” of medical education (Bleakley and Neilson 2024, p. 5).^3^

However, as one of us has argued, distaste for ambiguity is not necessarily bound to any genre, but is instead a hallmark of traditional literary scholarship that “seeks to derive [from texts] interpretation, metaphor, hermeneutic unpacking – in a word, knowledge” (Katz 2022, p. 30). Stephen Best and Sharon Marcus call this “symptomatic reading” that “t[akes] meaning to be hidden, repressed, deep, and in need of detection and disclosure by an interpreter” (2009, p. 1). Consider Hurwitz, who writes regarding patient narratives: “I experienced an epiphany of sorts—an abrupt and powerful sense that each of these stories had a singular medical subtext which I, as the general practitioner, must try to decipher” (2000, p. 2086). He reiterates Montgomery Hunter’s connection between detectives and doctors (pp. 2086–2087), and writes that both are engaged in a process of interpretation through narrative. But as far back as the nineteenth century, in the heyday of the theory of the novel that gave us the very idea that novel-reading helps us to unpack bodies in the real world, the doctor and detective were foils for real human connection—from the villainous vivisectionist Dr. Nathan Benjulia of Wilkie Collins’s *Heart and Science* who is willing to vivisect an innocent patient, to the bumbling Sergeant Cuff of Collins’s *The Moonstone* whose confidence in his own powers as a detective result in a suicide of an innocent woman he accuses (Katz 2022). Tying poems and patients to a chair and beating them with a hose to get them to confess their meaning is hardly productive at best, and can cause harm at worst (Collins 2006, p. 56). In the narrative humanities, for example, Jones and Tansey (2015) celebrate narrative’s ability to cultivate “skills that can be described as hermeneutic; the skills of interpretation, making sense of things” (p. 32). Even in defense of lyric poetry, Kirmayer falls into the language of interpretation: poetry can “confe[r] meaning and value on affliction” by “reframing experience as part of a narrative that gives it a larger meaning” (2024, p. 27). This kind of reading races past the surface to the hidden meaning beneath.

To read narrative as fragmented, affective, structured by uncertainty rather than archetypes ready for interpretation, opens up space for more affective and storytelling potentialities. Shapiro et al. (2009) get this partially right when they call for “curiosity about other narrative typologies,” and list: journey narratives, witnessing narratives, transformational narratives (p. 196). But what of narratives of failure? Of pain? Of unresolved deterritorialization, of pure affect? These narratives are more metaphysically troubling than they might first appear, for the romantic narrative reaffirms what Bishop calls “an anthropology … that a human is the animal-machine in need of something else” (2008, p. 19; see also Agamben 2005). Narrative restoration creates a hermetic space for hermeneutics by placing illness in literary quarantine from which *the human* emerges victorious through the process of meaning-making through interpretation. Without interpretation, the unspoken fear might well be, illness and pain reduce us merely to animality, but this assumes that (1) interpretation is the only way to make meaning, and (2) meaning-making is always a rational process that reinforces archetypal identities.

But *complex* meaning-making better occurs through embodiment. It is tempting to turn this into something like a two-step process: bodies connect, which allows them to interpret each other, and then we find meaning. Consider empathy, for example. The foundational work of Montgomery Hunter persists in the medical humanities: Hunter (1996) writes that “literature is unmatched for the access it gives to the experience of others, especially the inner lives of patients and the *meaning* of circumstances physicians cannot (or do not yet) share” (p. 312). Chiavaroli (2017) celebrates “the epistemological value of empathy (in the context of a humanities programme in medicine)” (p. 19); Kottow and Kottow (2002) write that “a well-read physician may increase his sensitivity in understanding illness and suffering” (p. 41). But as Arthur Frank writes, physicians taking his medical history “suffered from an illusion—that what they were learning about me was equivalent to knowing me as a person” (2014, p. 15). Haraway (1990) “cautions against a representational practice that transfers power from the represented to the author of that representation” (Garden 2015, p. 79); to confuse narrative with easy access to knowledge of a person inflicts a kind of violence against them that ossifies and turns people into mere symbolic puzzles that only the knowledgeable physician can interpret. Complex meaning-making, rather than facile interpretation, requires medicine to “tolerat[e] uncertainty and ‘wicked problems’ that cannot be solved but must be faced” (Bleakley et al. 2024, p. 2). This is why, though it does no favors for the status of the humanities in culture more broadly, contemporary literary studies is equally skeptical that interpreting a narrative brings one closer to “knowing” a person as a person (Keen 2007).

While it is a mistake to assume that narrative brings knowledge, it can, in a Cavellian sense, open up room for acknowledgement (Cavell 2002): knowledge says “I already know,” while acknowledgement says, “tell me.” Often the story of illness that emerges from “tell me” is fragmented, destabilizing, unsettling—and should be respected as such. Surface reading liberates narrative from the metaphysics of usefulness, but it also exposes patients and practitioners to the naked vulnerability that story requires and elicits. The Billy Collins poem to which we alluded earlier, “Introduction to Poetry,” contains moments of beauty and freedom on the surface:

I ask them to take a poem

and hold it up to the light

like a color slide

…

I want them to waterski

across the surface of a poem

waving at the author's name on the shore.

Attending to the surface illuminates the colors of the slide, the complex infinity of a water surface (Kornbluh 2019; Katz 2023). But it also suggests the unsettlingness of the surface. Collins asks his students to:

press an ear against its hive.

…

I say drop a mouse into a poem

and watch him probe his way out,

or walk inside the poem’s room

and feel the walls for a light switch.

There is, we suggest, something not entirely comfortable here. An ear pressed against a hive is unsettled by the buzzing, crawling, clicking of insects. A mouse dropped into a poem may be exploring, or it may be trapped, caged within a fragmented narrative that does not have a way in or out. Stumbling around in the dark of a narrative, one may never find the light switch. Yet, one may also have no choice but to try.

In the face of destabilization, it is unsurprising that one might seek coherence and sense-making. Speaking from experience: to receive a diagnosis and treatment plan for my (Katz) disability brought physical as well as emotional comfort. It is no wonder, then, that:

all they want to do

is tie the poem to a chair with rope

and torture a confession out of it.

They begin beating it with a hose

to find out what it really means.

Confronted with the disruptiveness of illness, or the impersonal maze of the hospital (physically and metaphorically), patients and practitioners may well want to tie the narrative of illness to a chair and beat a confession out of it.

Desperately wanting to make meaning out of suffering is part of the lived experience of being a material, animal body, a network of social and biological flows. It is far more authentically “human” than the coherent individual demanded by the torturer—in part because bodies connect and disconnect, relate and fall apart in fits and starts, not in coherent narratives with romantic structures. Illness forces one to be the mouse dropped in a poem, stumbling in the dark, pursued by the buzzing of the complex hive of material interrelations. Attention to those fits and starts, the next section will argue through Franz Kafka’s *The Trial*, cultivates embodied sensibility over sense-making.

Bishop cautions: “the usefulness of humanism is precisely about efficient control of the bodies—the animality—of the body politic” (2008, p. 22). A medical posthumanities approach to narrative relinquishes this control. Fitzgerald and Callard (2016) call for more space for affects of “ambivalence, awkwardness, frustration, failure, and so on,” an approach to narrative that “ ‘stays with the trouble’ of these relations and differences” between bodies, between patients and practitioners (pp. 40, 42). These troubles are part of being material bodies, and, as Evans (2016) argues, “no critique of medical reductionism can be adequate that does not begin from this recognition of our materiality” (p. 352). Messiness over coherence, surface over interpretation, destabilization over sense-making enables a medical posthumanities to bring practitioners closer to the intrinsic goods associated with narrative.

If the medical humanities uses narrative as just an instrumentally useful way to algorithmically interpret bodies, it will inevitably produce the same narratives and affects over and over: reiterated archetypes of the good death, the good patient, the good doctor. The medical romance is an interpretive frame that constrains the space of possible meaning-making to a set of affects and archetypes amenable to the genre. Outside gradual recapture of “the human” through interpretation lies in a zone of indifference, one that narrows the focus of medical narratives to more and more introspection. Dig deeper, interpret more, the medical romance says, and you will find meaning. If instead we understand narrative through surfaces, the kinds of affects and becomings that emerge make room for fits and starts, for expanding the zone of interest to include fragmented and even unsatisfying modes of embodiment and narrative trajectories—in a pair of words, for lived experience.

## The Trial: grasping for narrative

What is the core drama of Kafka’s great novel *The Trial*? It is Joseph K. in search for a narrative. To make meaning of his situation, K. must scramble to sort an open-ended set of continuously unravelling events. Despite his efforts, K. is unable to make even the slightest sense of what has happened to him, to the extent that he never even learns what he has been charged with. Indeed, much like one of Collins’ students, K. wishes he could simply beat the correct narrative out of the situation with a hose. And who could blame him for such a desire? He is certainly the mouse who has been dropped into a maze and now must find his way out.

“Several times already he’d wondered whether it might not be a good idea to draw up a written statement and submit it to the court. His intention was to present a brief account of his life, explaining for every event that was in any way important why he’d acted as he had, whether he now looked on his course of action with approval or disapproval, and the reasons he could adduce for either conclusion” (Kafka 2009, p. 80).

But, of course, K.’s defense never comes. He does not ever even succeed as far as learning the charges that are to come against him.

*The Trial* is a tangle of simulacra masquerading as signs in a way that exposes the instability of all signs (Troscianko 2014). If narrative in the medical humanities aims to craft a coherent narrative out of destabilization, *The Trial* baits readers into the same desire: to make sense of it all. But, as Baudrillard writes of the simulacrum: “It is no longer a question of imitation, nor duplication, nor even parody. It is a questioning of substituting the signs of the real for the real” (Baudrillard 1983). The narrative seems to beg for interpretation, but it is a trap; it elicits the feeling of the sign—as if it would all make sense, provided we (or Josef K.) could just tie it to a chair and beat it with a hose. Carceral affects circulate through the novel: K. is arrested; he is imprisoned in a bureaucratic nightmare; we, the readers, are trapped there with him; the sign is a cage and the signifier a key always just out of reach if we could just see below the surface. But, as with the arguments about lyric poetry, K. cannot manage to make the narrative into a coherent temporal sequence; it is a tangle of ambiguous spaces, a maze of affects and bodies.

But interpretation offers no acquittal. Instead, little moments of bodily fits and starts penetrate the tangle of almost-meaning. These embodied encounters are always just beside meaning-making, but resist even being deployed as a way to “understand” characters. When Josef K. is initially arrested, in the middle of a bumbling attempt to explain what is happening, Franz, who seems to be in charge of the arrest, “pat[s Josef] on the shoulder several times,” and then gives Josef a “probably significant but incomprehensible look” (p. 8). Incomprehensibility reigns, for Franz cannot offer him an explanation; instead, all he can offer is a brief embodied connection in a patted shoulder. Josef, though, remains desperate to make sense of it all. The narrator says that K. was happy to find himself “in the presence of a rational person” when he meets Franz’s supervisor, but the conversation is nonsensical (p. 12). The supervisor ultimately tells him, “Even if you had only said a few words, almost everything you have just said could have been deduced from your behaviour” (p. 13). Instead, the only moments of “sense” are those that are embodied in the senses: “Whenever K started shouting [the guards and Franz] became calm, almost sad, confusing him or, in a way, bringing him back to his senses” (p. 11).

The story is filled with grasping or *fassen*—a desperate, animal endeavor to compel or connect. In a moment where K. might have been able to find an account that might make sense of the madness, he can only grasp it, claw-like: “I am not afraid of this legal account book, even though it is a closed book to me since I can only bring myself to touch it [*anfassen*] with the tips of two fingers” (p. 34). Later, K. takes [*fasste*] a defendant by the arm with two fingers (p. 51) in an attempt to connect with the man. But, despite his ostensibly gentle grasp, “the man cried out as if K. had touched his arm not with two fingers but with red-hot pincers.” (p. 51). Frustrated, K. “grasped [*fasste*] him really firmly, pushed him back down on the bench, and went on” (p. 51). Later, when the guards are being punished, Franz comes to Josef and grasps his arm (p. 60).

Even K.’s grasping of women, while equally strange, does not produce the perhaps expected patriarchal grounding of meaning through the control or mediation of women’s bodies. In his bizarre encounter with Fraulein Bürstner:

He went out, grasped [*fasste*] her, kissed on the lips and then all over her face, like a thirsty animal furiously lapping at the water of the spring it has found at last. Finally, he kissed her on the neck, over the throat, and left his lips there for a long time (p. 26).

But though he leaves “satisfied,” he is “surprised that he wasn’t even more satisfied” (p. 26). Bürstner is far less satisfied: nodding “wearily,” she allows him to “kiss her hand, though she had already half turned away, as if she were unaware of it, and went into her room, shoulders drooping” (p. 26). The expectation is further subverted in his later encounters with women. The woman who suggests an affair successfully “clasp[s] [*fasste*] K.’s hand” (p. 43), but when he attempts to “gras[p] [*haschte*] at her hand involuntarily,” he finds only “empty air” (p. 45). In his sexual encounter with Leni, *her* hand is “a pretty claw” (p. 78). When he “put his arms round her [*umfasste*] to stop her falling”—which might seem like a dominant comportment of his body—instead, he is pulled down atop her and she decrees, “Now you belong to me” (p. 78). Later, she grasps [*fasste*] his arm and tries to pull him out of the lawyer’s room (p. 131), and when he grasps [*fasste*] her in turn, it “wasn’t love that made him hold it tight” (p. 137).

To grasp, as K grasps women, is to take hold of, to control as a dominant agent. But throughout the trial, K.’s endeavors to grasp begin to fail, and he instead is grasped. As he attempts to leave the room where he gives his speech, someone grabs [*fasste*] his collar and prevents him from moving (p. 38). Even the young boy who leads him to give his first speech grasps [*fasste*] his hand (p. 31). And this grasping begins to undermine his ability to navigate the process, and starts to compel him along with the flows of power: Leni “clasp[s] [*umfasste*] her hands behind his neck” as she tells him that he must confess (p. 77). Most importantly, he can be grasped by intangible forces and flows; when he finds the surreal room where the guards are being beaten, K.’s “curiosity got the better of him” or more accurately <<dann aber *fasste* ihn eine derart unbezähmbare Neugierde>>: he is grasped by an indomitable curiosity (p. 58). And in the very end, he cannot grasp: “K knew very well that it would have been his duty to grasp [*fassen*] the knife himself as, going from hand to hand, it hung in the air above him and plunge it into his own body” (p. 164)—but he cannot, and his last words, importantly, are “Like a dog!”, as if he has been stripped of all human agency.

It is tempting—a temptation we fell into, in fact, before we went to the original German—to want to read the physical grasping as a mirror of K.’s inability to “grasp” what is happening to him. K.’s uncle cries, “You always had such a clear grasp of things, has it deserted you now, of all times?” (p. 68). The painter tells him, “Now you have grasped the crux of the matter” (p. 151). But neither of these translated “grasps” are actual grasps; the word does not work that way in German. Having sat *The Trial* down with the guards and beaten it with a hose, it laughs at the notion that it would have a secret to give up.

The “secret” is right there on the surface: that this is a book filled with flows of physical power and bodies being moved and penetrated and contained and dragged about in ways that they often do not themselves understand. The process itself is mystifying, but it is all the more powerful not because it is difficult to conceptually grasp, but because the nonconscious, involuntary grasping between bodies undermines the hollow sense of decorum underneath. The judge does not sit with “peace and dignity,” but rather, “clasp[s] [*umfasste*] the back and arm of the chair firmly … as if any moment he was about to leap up with a vehement and perhaps outraged gesture” (p. 76). He is, in many ways, the most honest body in the novel: a body that is meant to stand for abstract concepts, but is instead clinging desperately in preparation to lash out with physical movement.

## In the presence of the examining magistrate: constructing a zone of interest/indifference

Joseph K. reaches out and grasps. Is not, then, K. the architect of his own actions? It occurs to K., for whatever reason (whether due to his own base urges or some other motivation), to seize Fräulein Bürstner, and forcefully kiss her. He is rightly held responsible for these actions and the actions can be credited to him. When we examine what happened at that moment, we do not seem to need recourse to other factors to explain why these events have transpired. Not long after K. seizes Fräulein Bürstner, he himself has a chance to reflect on what transpired:

A short time later K. was in bed. He quickly fell asleep, but first he thought for a while about the way he had behaved; he was satisfied with it, but was surprised that he wasn’t even more satisfied; he was seriously concerned for Fräulein Bürstner because of the captain (Kafka 2009, p. 26).

The encounter with Fräulein Bürstner is nicely contained within K.’s own estimation of the situation and how he has chosen to act in response to it.

Now we, as outside observers, can ask further questions about K.’s behavior. Why did K. believe (even implicitly) that forcefully grabbing and kissing a woman is an appropriate way to act? Perhaps it’s because his father raised him that way by modeling that behavior for him, because he was born in a time where patriarchal standards dubbed such actions as acceptable, or because he himself has developed derogatory attitudes towards women. In the text itself, we aren’t given access to K.’s own life events, so we cannot directly adjudicate. One may wonder if it even matters from where K. has derived these beliefs. What matters is that K. now acts in this way. The personal characteristics that shaped his interaction with Fräulein Bürstner are contained within his skin, so to speak. These “outside factors” are negligible in the context of this particular encounter. K. himself is not sufficiently arrested by them to delay his falling asleep.

Indeed, what if K. were to ask such questions? Why not press on further? If his attitudes came from his father, why not regress further and ask where his father got them? K. could certainly do this. Yet, there must be some point at which K. quits his search and decides he knows enough to give an account of why he has acted how he has acted. The end to searching must occur even if for no other reason than a practical concession to the demands of time passing. There must be some things that will not figure into his account, whether he has realized this or not, and whether it is a good idea to ignore these things or not. This outside space may be named a *zone of indifference*. It names that space outside of the core *zone of interest*. The zone of interest delimits those things that the questioner attempts to take account of, find the meanings of, interpret, etc. Laboratory science, for instance, is premised on this distinction. By taking a thing from its natural context, you attempt to put it in an artificial environment whereby the zone of indifference is everything outside of that laboratory. This makes a good deal of sense in many cases and has obviously been a useful method of investigation. In the case of narrative, things become much more complicated.

There are two motifs in Kafka’s navigation of the zones of interest and indifference in *The Trial*. The first is the realization that the zone of interest contained previously undetected, but pertinent, meanings. The second is the realization that something previously unknown (and hence in the zone of indifference by default) is actually so important that it fundamentally shifts K.’s entire interpretation of the situation. The loss of narrative integrity can come from either direction. For the first motif, we can look to his interactions with Leni. Huld tells K. that Leni is attracted to him only because of imperceptible physical changes that happen to an accused man. If only he had been able to look deeper, he would have perhaps detected those changes.

On the other hand, the interruption of the zone of indifference can be spotted in K.’s interrogation in front of the examining magistrate. The chapter is among the book’s most bizarre, as K. is jammed into a room so full to the brim with spectators that the attendees “had brought cushions, which they placed on their heads so as not to hurt them as they pressed them against the ceiling” (Kafka 2009, p. 32). Although the room is itself hidden away in an unremarkable building in a decrepit part of town, it turns out to be an important court office where K. is to be publicly interrogated.

For K.’s own part, he certainly embodies the spirit of Billy Collins’ above-cited poem; K. is a mouse who has been dropped into a maze and must now find his way out. All around him are strange and difficult to understand signs, such as the on-again-off-again applause from the crowd and the secret signals passed from the magistrate to audience members. His defense he offers for himself is as much aimed at exculpation as well as understanding. At various moments he seeks to prove himself innocent, to speak aloud what he believes has happened to him, to decipher the crowd’s attitudes, to shape their opinion of the magistrate, and, finally, to uncover how the whole system operates. We see this latter motive most clearly in a particularly dramatic scene:

Silence immediately fell, such was K.’s control over the meeting. They were no longer all shouting at once, as they had at the beginning, they were not even applauding any more, but they seemed to have been convinced already or were well on the way to it.

‘There is no doubt—’ said K. very quietly, pleased with the way the whole assembly had pricked up their ears in expectation; in the silence a buzzing arose which was more exciting than the most ecstatic applause. ‘There is no doubt that there is a large organization at work behind this court’s every operation, in my case the arrest and today’s examination. An organization which not only employs venal guards, foolish supervisors, and examining magistrates who are at best unassuming, but which, beyond that, doubtless maintains a bench of judges of high, indeed the highest standing, with their inevitable numerous entourage of ushers, clerks, police officers, and other assistants, perhaps even, I do not hesitate to use the word, executioners. And the point of this large organization, gentlemen? It consists in arresting innocent persons and instituting pointless and mostly, as in my case, fruitless proceedings against them. How would it be possible, given the pointlessness of the whole business, to avoid the worst kind of corruption among its employees? It is not possible. Even the most senior judge could not guarantee it in his own case (Kafka 2009, p. 37).

One gets the sense that these thoughts are spilling out of K. quite beyond his control. His case is, in a sense, undecidable because the true forces that shape it are so distant, so alien, that not even the most senior judge could fight against it.

Yet, in a sudden reversal, K. at once observes a set of military badges hanging from the jackets of those in attendance, exclaiming:

‘So!’ K. cried, throwing his arms in the air — this sudden insight needed space — ‘You’re all officials, as I see, you’re the corrupt gang I was inveighing against, you crammed into this hall as eavesdroppers and snoopers, pretending to be two different parties, and one applauded, just to test me; you wanted to learn how to seduce innocent people (Kafka 2009, p. 39).

It turns out that what K. hypothesized as operating outside of the room actually was sitting in front of him this whole time. What he took as an appeal to his fellow citizen over and against the need to concern himself with the operations of this “large organization” is proven to be quite mistaken.

## The posthumanities and the breakdown of medical romance

### Physician-assisted suicide

K.’s case is certainly unique for its severity. The degree to which what is outside the room forces its way inside is remarkable, and K.’s own narrativizing is unusually agent-like – the narrative itself has an outsized ability to shape K., the spectators, and the magistrate as an autonomous entity. Yet, these dynamics from *The Trial* can be found in real-world scenarios as well. By way of concluding this paper, we will return to decision-making and narrative as they manifest in the decision to request or refuse physician-assisted suicide (PAS) in the case of disabled patients.

As a term, PAS is polyvalent, potentially signifying several forms of active or passive euthanasia. Any version will have a multitude of defenders and detractors. These defenders and detractors are themselves ideologically diverse crowds. PAS has variously been called a way to alleviate interminable suffering (Angell 1997), the killing of innocents (John Paul II 1995), and/or a manifestation of respect for patient autonomy (Dworkin 1994). It’s a locus of the culture wars, a political issue, and also a deeply personal one.

We see also in PAS the possibility for medical romance *par excellence*. The practice is unavoidably tied up with foundational moral concepts and so offers its perpetrators and recipients the possibility of participation in narratives surrounding fundamental values. Indeed, PAS as a practice has sometimes been called “death with dignity,” and who, after all, would not want to die with dignity? Dignity, in turn, stands alongside other possible values. Think, for instance, of Henrik Ibsen’s *Hedda Gabler*, where the protagonist Hedda encourages her former lover Eilert to take his life with a pistol that she has lent him. Just before she gives him the gun, she implores him: “Eilert Lovborg-listen to me.--Will you not try to--to do it beautifully?” (Ibsen 2003) Upon learning of Eilert’s death, Hedda remarks that “there is beauty in [it]” and that he “had the courage to do--the one right thing.” Under her breath, she mutters to herself: “Oh, what a sense of freedom it gives one, this act of Eilert Lovborg's.” And, before the illusion is broken, she rather ghoulishly claims:

“I only know that Eilert Lovborg has had the courage to live his life after his own fashion. And then--the last great act, with its beauty! Ah! that he should have the will and the strength to turn away from the banquet of life--so early” (Ibsen 2003).

Hedda would then be told that Eilert seems to have accidentally fired the gun in his breast pocket while arguing with a prostitute, shooting himself through the stomach and dying a distinctly un-beautiful death.

Ibsen has given us a proto-bioethics of PAS in these passages. Although there is nothing physician-assisted (or even purposeful) about Eilert’s death, the means by which she appraises it as an act of courage, freedom, beauty, and strength, are not alien to modern bioethics. Indeed, these means of appraisal form part and parcel of modern narratives surrounding PAS. Though, what makes PAS most notably different from Eilert Lovborg’s suicide is the position of the sick, ailing, and/or terminally-ill patient who requests PAS. Unlike the passionate Eilert who takes his life when there is still so much possible to reap from the “banquet of life”, PAS is most typically thought of in relation to people for whom the banquet holds few future pleasures and precious little time to look for them.

All the same, the cases are not so dissimilar. Let us look, for instance, to the following passage from Nietzsche:

A sick person is a parasite on society. Once one has reached a certain state it is indecent to live any longer. Vegetating on in cowardly dependence on physicians and their methods, once the meaning of life, the right to life has been lost, should be greeted with society’s profound contempt. The physicians, for their part, ought to convey this … Die proudly if it is no longer possible to live proudly (Nietzsche 1998, p. 61).

Nietzsche’s exhortation to the parasitic invalid to die proudly is not so different in form to Hedda’s glee that Eilert has chosen to die courageously. The meaningful difference would supposedly lie in the *reasons* one chooses for dying. On Nietzsche’s telling, the invalid’s decision to die stems from the indecency of being dependent on physicians/society. Regardless of how we may evaluate it, this is certainly a different motive that Eilert’s grief at the loss of his irreplaceable manuscript.^4^

### Disability studies and physician-assisted suicide

What separates PAS from simple medical murder is that PAS is requested by a patient. And, for all but the strongest defenders of the right to free and capricious autonomy, that patient’s request must be a “good one.” In other words, there are situations under which one requests PAS where it would be justified (or even obligatory) to refuse that request. For PAS’s defenders, there must be something in the mind of the patient that drives them to request PAS, and that thing must be a good reason to request PAS.

What should we think in the case that a disabled patient requests PAS on the grounds of their disability being intolerable? This question has been an important point of analysis for scholars in disability studies. For instance, there is certainly a medical double standard with disabled people and suicide prevention. The default behavior of medicine towards suicidality is to try to prevent suicide. However, advocacy groups such as Not Dead Yet have noted that this is *not* the default behavior towards many disabled people:

“Not Dead Yet's central argument is that legalized assisted suicide sets up a double standard for how health care providers, government authorities, and others respond to an individual's stated wish to die. Some people get suicide prevention while others get suicide assistance, and the difference between the two groups is the health status of the individual. As explained in the brief submitted by Not Dead Yet, et al. in the Montana case, this is a clear cut violation of the Americans With Disabilities Act (ADA). Quoting at length from the Not Dead Yet brief:

*Assisted suicide singles out some people with disabilities, those labeled “terminal” or very severely impaired, for different treatment than other suicidal people receive …For certain people who are disabled, suicide will be viewed as understandable and acceptable. According to assisted suicide advocates, an incurable disability is sufficient for eligibility*” (Coleman 2010, pp. 43–44).

A pernicious double standard indeed, and one that socially derives from discriminatory beliefs about disabled people. Ackerman (2018) notes with distaste Dworkin’s (1994) invocation of the above-cited Nietzsche passage. Dworkin believes that a patient’s view of themselves as a sick and dependent parasite are reasonable grounds for that patient to wish to die. On Ackerman’s take, this lays the double standard of suicide prevention in full view.

Yet, the hard questions about PAS for disabled people remain. Could a disabled person’s life actually be sufficiently intolerable for PAS to be warranted, even if we account for discriminatory beliefs in healthcare? Or, alternatively, are there cases where a disabled person requests PAS where that request ought to be honored? The most straightforward argument for the affirmative stems from a respect for patient autonomy, which prominent disability rights advocates such as the late Andrew Batavia have advocated for (Batavia 1997). Batavia of course recognizes the existence of anti-disability discrimination, but nevertheless holds that the autonomous choice to seek PAS is possible and respectable.

We have before us then a true ethical dispute, but also one which manifests with narrative dimensions. As we mentioned, PAS allows for the telling of medical romance like few other moments in healthcare. If medical romance is about the preservation of personal identity, then PAS allows for the most dramatic instances of preservation of personal identity at the expense of one’s body, and indeed one’s own life. The difference between Nietzsche’s invalid and Eilert Lovborg seem to slip away as both cases offer their respective parties the chance to assert their personal identities in one final, terminal, grand, romantic, self-destructive gesture. How we should evaluate PAS for disabled patients seems at least partially clear, then. The pertinent questions are: what sort of story is a patient telling about themselves when they request PAS—and should we approve such stories?

Yet, the strength of medical romance is also its danger. That one *could* look at the situation in this way is certainly true. However, as with Joseph K., there are moments where important meanings cannot be uncovered only via the means of further introspection. In other words, the borders of the zone of interest corresponding to this particular narrative framing foreclose on pertinent meanings and interpretive potentials. This fact is particularly ironic when the demand for further introspection does not stem from the patient themselves.

For instance, consider Longmore’s (2005) treatment of the cases of David Rivlin and Larry McAfee. Both of these men were quadriplegics who were dependent on mechanical ventilation and who successfully petitioned the court for physician-assisted suicide. Rivlin would go on to complete suicide whereas McAfee would eventually regain his will to live after his quality of life improved. The public discourse surrounding these men focused on such features as Rivlin’s “cruel semblance of life” (Dougherty and Tessler 1989, p. 56, cited in Longmore 2005) and the rationality of McAfee’s decision to end his own life. Indeed, the judge presiding over McAfee’s case “declared his admiration for McAfee’s [courage]” (Longmore 2005, p. 44).

These points can motivate either a disability rights critique of the double standard for suicide prevention in disabled people or a disability rights support of autonomous decision-making for disabled individuals. However, Longmore invites us to look further. I mentioned earlier that McAfee ultimately decided against going through with the PAS that he was legally entitled to. Why? Why did McAfee’s quality of life improve so dramatically? The answer lies in distinctly non-romantic forces: the way health insurance pays out benefits for disabled people. In the case of McAfee, this meant that the State of Georgia would pay for McAfee to stay in an ICU or in an intensive nursing care facility, but would not pay significantly more modest sums for him to receive some quality of independent, at-home care. As Longmore puts it, “the problem was not reimbursement of health-care providers but the state’s failure to fund independent living” (Longmore 2005, p. 42). As a result, McAfee may have suffered from ICU psychosis, but *certainly* suffered from being kept in facilities where he spent his hours “watching the clock, watching TV and with a small amount of reading... There is nothing to look forward to,” he said. “I wake up in the morning just fearful of each new day... There’s not much else to do but lie there and think of things” (Longmore 2005, p. 42).

In sum total, then, to adopt the framing of the medical romance for cases like Rivlin and McAfee is to confine oneself to narrative conventions that cannot find the right details to capture what is interesting about this case. Neither of these cases are much like an Eilert Lovborg who resolves to end his life beautifully, but rather are the stories of men whose interests have been overlooked by large, impersonal societal structures. As Braswell (2019) notes, the shape of the US Medicare Hospice Benefit plays an outsized role in decisions related to end-of-life care in the United States. Where people are practically allowed to seek care, under what contexts, and what that means for their quality of life is often largely determined by this institution. In the case of men like Rivlin and McAfee, this point is wholly lost in the legal proceedings.

Yet, is this not just substituting one romance for another? We claim not. Rather, to get at the importance of things like the Hospice Benefit is, in this case, to decenter the “woundable storyteller” who is implicated in the narrative. Importantly, the cases surrounding Rivlin and McAfee proceeded by questioning those things that were within the grasp of the autonomous decisions of these men. This puts things that do not respond to autonomous decisions, such as the structure of the Hospice Benefit, within the zone of indifference. It is rather thereby a preexisting feature of the decision-making process, known and relevant only for its effects on the zone of interest.

These effects, though, are quite large. As Braswell put it:

The dependence of US hospice care on existing networks of familial caregiving is relevant to the reasons why individuals in states where PAS is legal - most notably Oregon - choose to end their lives. Indeed, in 2014, 40% of Oregonians who petitioned for PAS mentioned a fear of burdening family members, friends, and caregivers as a motivating factor in their desire to die ... Such a fear is a product of the way that our end-of-life care system is organized. Terminally ill individuals will feel like burdens on their families in a system that places the burden of their care on their families. Where PAS is legal, individuals who do not want to burden their families may seek out the practice. A desire to die in such circumstances is understandable, but troubling. If suicidal ideation is, in such cases, partially a product of the organization of US end-of-life care, then it could be alleviated by improvements in the end-of-life care system. PAS may assist the suffering of these particular terminally ill individuals. But it leaves in place the underlying social structures that, in part, produced their suffering to begin with (Braswell 2018, p. 91).

This presents a new way to tell the story of patients who request PAS, and it suggests for society a way forward that does not rely on their approval or distaste for the decision to request PAS and under what contexts. Rather, to break out of the dominant mode of the medical romance, to shift the zone of indifference, is to realize the other dynamics “out there” in the world that shape the lives of these woundable storytellers in the clinic.

## Conclusion: what’s in a name? Looking towards the medical posthumanities

All together, then, we believe this breakdown of medical romance as the dominant narrative generic archetype serves as a contribution to the on-going call for critical dialogue between the medical humanities and posthuman theory enunciated so well by Crocker et al. (2023). Where the task of critical posthumanism is to interrogate the long shadow of humanity posited as “the sole autonomous agent” (Daigle 2023, p. 227), we believe that combatting the hegemony of medical romance aids in the need “to explore the complex entanglements of human, animal, and ecological health as a mode of knowledge construction” (Welch 2024, p. 12). For our analysis, this is borne out in a demonstration of how consideration of the zones of interest and indifference can indicate anthropocentric interpretive frames. While these are not always bad, neither should they be allowed to assume a default position.

This, then, is our contribution towards this dialogue: a recognition of the provisionality of interpretation and meaning in light of the continuously flowing possibilities of newly charted zones of interest and indifference. Such a project is neither a surrender to relativism nor does it foreclose on interpretive frames that isolate particular important dynamics in the world. Although meaning and interpretation are provisional, this does not mean that any meaning or any interpretation are as good as any other. The natural correlate of this observation is that it is a mistake to think of the humanistic framing of medical romance as by default as good as any other interpretation. Rather, the work of the medical posthumanities is to approach narrative with a righteous irreverence. It is both to blow apart as well as to build back up. Such a task preserves the importance of the humanities in both its critical and constructive modes. For, after all, posthumanism springs from the question “what does it mean to think of beings, deeply and fundamentally, in terms of interrelations, dynamic processes, and manifold entanglements?” (Daigle and Hayler 2023, p. 3) These entanglements, in turn, both nurture our narrativizing as well as resist it.

## Data Availability

No datasets were generated or analysed during the current study.

## Footnotes

1 For instance, Bleakley himself (2023) sees an important task of the medical humanities as fundamentally translational in nature, involving the explosion of crass instrumentalism in naive medical education into various new meanings.

2 As Bishop argues, when narrative medicine pits the human body, illness, healthcare systems against *the human*, it reiterates a “dualism of meaning and material,” which it then “link[s] together by the Western metaphysics of efficiency, which has objects standing in reserve waiting for human manipulation” (Bishop 2008, p. 18). The impulse to see narrative as committed to “connectedness and structure”—“properties medicine valorises in the construction of clinical cases,” Bates and Hurwitz write (p. 561)—imposes the metaphysics of efficiency onto narrative more broadly and patient narratives in particular. It turns them into efficient and effective tools to cultivate *the human*, to make illness and injury usefully sensible through “a useful and coherent story” as Launer advocates (Launer 2013, p. 147).

3 In place of uninspired narrative, Bleakley and their co-authors like Shane Neilson (2022) offer lyric poetry: absent of the temporal component of narrative (Kirmayer 2024, p. 27), designed intentionally to “interrupt or interfere with narrative” (Hirsch 1999, p. 242) to better “draw attention to neglected elements of experience” (Kirmayer 2024, p. 28). In place of the dead metaphors of narrative, Bleakley argues, lyric poetry’s metaphors “release the mind and body from the prison of the literal” (Bleakley 2017, p. 25). As Lakoff and Johnson (1999) argue, metaphor is fundamentally embodied; “language,” Bleakley writes, “is a tangible medium through which we experience and make sense of embodiment” (2017, p. 30).

4 Although it is outside of the scope of this discussion, it is worth noting that Hedda herself does view Miss Tesman’s decision to take in an invalid to nurse as being a burden. One could interrogate the links between her views about sickness, dependency, and her stance towards Eilert’s desire to kill himself.
